# A Multimodal Meta-Analysis of Structural and Functional Changes in the Brain of Tinnitus

**DOI:** 10.3389/fnhum.2020.00028

**Published:** 2020-02-25

**Authors:** Shirui Cheng, Guixing Xu, Jun Zhou, Yuzhu Qu, Zhengjie Li, Zhaoxuan He, Tao Yin, Peihong Ma, Ruirui Sun, Fanrong Liang

**Affiliations:** ^1^The Acupuncture and Tuina School/The 3rd Teaching Hospital, Chengdu University of Traditional Chinese Medicine, Chengdu, China; ^2^The First Affiliated Hospital of Chengdu University of Traditional Chinese Medicine, Chengdu, China

**Keywords:** meta-analysis, tinnitus, multimodal, neuroimaging, signed differential mapping, voxel-based morphometry

## Abstract

Brain imaging studies of tinnitus patients have revealed marked changes in brain structure and function, but there are inconsistencies in those findings. In this meta-analysis, we investigated concurrence across studies to clarify those abnormalities in brain structure and function in tinnitus. Neuroimaging studies published up to December 6, 2019 were searched in the PubMed, Web of Science, EMBASE, and Cochrane Library databases, Chinese Nation Knowledge Infrastructure, Chinese Biomedical Literature Database, the Chongqing VIP, and Wanfang Database. Study selection, quality assessment, and data extraction were performed by two independent researchers. Anisotropic effect size signed differential mapping (AES-SDM) was used to perform a multimodal analysis of available studies reporting whole-brain structural or functional data in tinnitus patients. There were 14 studies that met the inclusion criteria. The structural dataset comprised 242 tinnitus patients and 217 matched healthy subjects (HS), while the functional dataset included 130 tinnitus patients and 140 matched HS. Our analysis revealed structural alterations in the superior temporal gyrus, middle temporal gyrus (MTG), angular gyrus, caudate nucleus, superior frontal gyrus, and supplementary motor area, as well as functional differences in the MTG, middle occipital gyrus, precuneus, and right inferior parietal (excluding supramarginal and angular) gyri. The multimodal analysis revealed significant differences in the right MTG of tinnitus patients relative to HS. These findings suggest the involvement of the cortico-striatal circuits in the neuropathology of tinnitus.

## Introduction

Tinnitus is characterized by the perception of sounds in the ears or head in the absence of any external stimulus (Wegger et al., 2017; Bauer, 2018) and affects ~10–15% of the population (Shargorodsky et al., 2010; Baguley et al., 2013; Bauer, 2018). It often occurs in association with hearing impairment, insomnia, depression, anxiety, and difficulty in concentration, and significantly impairs patients' quality of life (Reynolds et al., 2004; Langguth et al., 2013; Zeman et al., 2014). Although phantom sounds are thought to originate in the cochlea, tinnitus is also related to maladaptive neuroplastic changes in the central nervous system as it often persists even after the auditory nerve has been severed (Berliner et al., 1992; Silverstein, 2001).

Structural and functional alterations in multiple auditory and non-auditory brain regions have been detected in tinnitus patients (Adjamian et al., 2009; Husain and Schmidt, 2014). For example, increased volume of gray matter (GM) in the bilateral superior temporal gyrus (STG), right middle temporal gyrus (MTG), and right supramarginal gyrus and decreased GM volume in the bilateral hypothalamus, left superior frontal gyrus (SFG), and right occipital lobe were observed in tinnitus patients (Boyen et al., 2013). On the other hand, some studies have reported no changes in GM volume associated with tinnitus (Landgrebe et al., 2009; Melcher et al., 2013). Furthermore, one morphometric study showed an increased GM volume in the right SFG (Schmidt et al., 2018), whereas the opposite was demonstrated by another voxel-based morphometry (VBM) study (Boyen et al., 2013). Besides anatomical changes, functional alterations have been detected in tinnitus (Chen et al., 2014, 2015; Han et al., 2018a), as evidenced by increased amplitude of low-frequency fluctuations (ALFF) in the right MTG, right SFG, and right angular gyrus and decreased ALFF in the left cuneus, right middle occipital gyrus, and bilateral thalamus (Chen et al., 2014). However, increased regional homogeneity (ReHo) in the frontal cortex (Chen et al., 2015) and decreased ReHo in the frontal gyrus (Han et al., 2018a) have also been detected in two different studies.

Although tinnitus is thought to involve many brain areas, the evidence on regional alterations has been inconsistent across studies (Boyen et al., 2013; Chen et al., 2015; Han et al., 2018a; Schmidt et al., 2018), possibly due to differences in sample size, demographic and clinical characteristics of patients, imaging modality, and analytical approach (Boyen et al., 2013; Allan et al., 2016; Schmidt et al., 2018). At present, little is known about the association between structural and functional changes, as there have been few multimodal studies. A previous meta-analysis of functional magnetic resonance imaging (fMRI), positron emission tomography, and single-photon emission computed tomography studies revealed an increased activity in several brain regions including right SFG, MTG, bilateral insula, inferior frontal gyrus, parahippocampal gyrus, and posterior lobe of the cerebellum and a decreased activity in the left cuneus and right thalamus (Chen et al., 2017). Cross-validation from structural and functional brain alterations is still required to confirm the reliability of these findings and better understand the cerebral characters of tinnitus.

Anisotropic effect size signed differential mapping (AES-SDM) is a method used for the meta-analysis of neuroimaging data. Unlike activation likelihood estimate and multi-level kernel density analysis that are based on regional likelihood or frequency of reported peak locations of significant activation clusters (Wager et al., 2007; Eickhoff et al., 2009), AES-SDM combines peak coordinates and statistical parametric maps and uses standard effect size, which allows an exhaustive inclusion and accurate estimations of studies (Radua and Mataix-Cols, 2009; Radua et al., 2012a, 2014). AES-SDM has high sensitivity, consistency, and a low rate of false positives (Radua et al., 2012a) as well as higher accuracy than other coordinate-based methods owing to increased reliability and validity of the neuroimaging data (Radua et al., 2012a).

We speculated that there is an overlap in the structural and functional changes in patients with tinnitus compared to healthy subjects (HS). In this study, we used AES-SDM to test this hypothesis by comparing GM volume and spontaneous activity reported in whole-brain VBM and fMRI studies.

## Materials and Methods

### Search Strategies

A systematic search strategy was used to identify relevant studies. Two independent researchers (SC and JZ) performed a two-step literature search of the PubMed, Web of Science, EMBASE, and Cochrane Library databases, Chinese Nation Knowledge Infrastructure (CNKI), Chinese Biomedical Literature Database (CBM), the Chongqing VIP (VIP), and the Wanfang Database (WF) to identify structural or functional imaging studies on tinnitus. The search was conducted up to December 6, 2019, with no time window specified for date of publication. The following English search terms were used: (“tinnitus” OR “acousma” OR “akoasm” OR “phantom sound” OR “auditory phantom sensation”) AND (“magnetic resonance imaging” OR “MRI” OR “functional MRI” OR “fMRI” OR “voxel-based morphometry” OR “VBM” OR “voxelwise” OR “gray matter” OR “regional homogeneity” OR “ReHo”) AND (“resting state”). This search strategy has been modified to be suitable for Chinese electronic databases. The reference lists of articles cited in reviews were manually checked for any studies not identified by our database searches. Corresponding authors were contacted by email or telephone if data were not available or if questions about the data arose.

### Selection Criteria

The included studies met the following criteria: (1) original paper reported in English or Chinese viewed journal; (2) comprised patients diagnosed with subjective tinnitus and HS; (3) involved whole-brain structural or functional imaging of both groups; (4) results of x-y-z coordinates were reported in Montreal Neurological Institute (MNI) or Talairach coordination; and (5) a 1.5T or 3.0T MRI scanner was used.

The exclusion criteria were as follows: (1) it did not report a VBM comparison of GM volume, modulated GM concentration, ReHo between tinnitus patients and HS; (2) only reported region of interest (ROI) findings or small volume corrections in pre-selected ROIs; (3) data were already covered in one or more articles that were included in our analysis; (4) the patients had other uncontrolled diseases including liver or kidney or hematopoietic diseases, endocrine and immune diseases, mental disorders, malignancy, etc.; and (5) recruited participants were not adults. Studies of pulsatile tinnitus were also excluded owing to its unusual mechanism (Liu et al., 2018).

Authors of studies in which MNI or Talairach coordinates (necessary for voxel-level quantitative meta-analysis) were not explicitly reported were contacted to reduce the possibility of a biased sample set. In cases where the same or similar datasets were used in separate papers, only data from the analysis of the largest sample were included.

### Recorded Variables

The following information was extracted from the selected studies: first author, year of publication, sample size, number of female participants, the mean age of participants, duration of tinnitus, type of MRI (1.5 or 3.0 T), image package, and full width at half maximum (FWHM). Statistically significant coordinates including the direction of GM volume or differential activity between tinnitus patients and HS were extracted. This information is shown in Table 1.

**Table 1:** Demographic and clinical characteristics of subjects in MRI datasets included in the meta-analysis.

**Study**	**Analysis**	**Number (female)**		**Mean age (year)**		**Durations (month)**	**Hearing loss**	**MRI scanner**	**Software**	**Smoothing (FWHM)**	**P value**	**Correction method**	**Quality score**
		**TIN**	**HS**	**TIN**	**HS**	**TIN**							
**VBM STUDIES**													
Mühlau et al. (2006)	VBM	28 (15)	28 (15)	40	39	53	No	1.5T	SPM2	8	<0.05	FDR	10
Landgrebe et al. (2009)	VBM	28 (13)	28 (13)	32.2	31.2	53.3	No	1.5T	SPM2	10	<0.05	FDR	10.5
Boyen et al. (2013)	VBM	31 (11)	24 (8)	56	58	12–348	Yes	3.0T	SPM5	8	<0.05	FWE	10
Melcher et al. (2013)	VBM	24 (12)	24 (12)	46.9	45.8	4.8–480	Mixed	3.0T	SPM8	8	<0.05	FWE	9.5
Krick et al. (2015)	VBM	22 (9)	22 (9)	42.6	NA	1.08	Mixed	3.0T	SPM8	10	<0.05	FWE	9.5
Allan et al. (2016)	VBM	73 (30)	55 (25)	58.38	56.91	NA	Mixed	1.5/3.0T	SPM8	10	<0.05	FWE	10.5
Schmidt et al. (2018)	VBM	15 (5)	15 (6)	55.13	52.93	>12	Mixed	3.0T	SPM12	10	<0.05	FWE	10
Han et al. (2018b)	VBM	21 (12)	21 (12)	44.1	43.5	20	No	3.0T	SPM8	8	<0.01	AlphaSim correction	10
**ReHo STUDIES**													
Jin et al. (2011)	ReHo	20 (12)	20 (13)	41	49.1	NA	Mixed	1.5T	SPM2	NA	<0.005	Uncorrected	8.5
Yang et al. (2014)	ReHo	18 (4)	20 (5)	43	42	16.8	Mixed	3.0T	SPM5	NA	<0.05	FWE	9.5
Chen et al. (2015)	ReHo	29 (13)	30 (15)	40.9	46.2	39.5	No	3.0T	SPM8	4	<0.01	AlphaSim correction	10.5
Cai et al. (2017)	ReHo	10 (6)	10 (6)	39.6	NA	NA	Mixed	3.0T	Dpabi	4	<0.001	TFCE	8.5
Han et al. (2018a)	ReHo	25 (15)	25 (15)	44.64	43.96	14	No	3.0T	SPM8	4	<0.001	AlphaSim correction	10.5
Han et al. (2018b)	ReHo	21 (12)	21 (12)	44.1	43.5	20	No	3.0T	SPM8	4	<0.05	AlphaSim correction	10
Fan (2018)	ReHo	7 (2)	14 (4)	40.86	48.50	>3	Mixed	3.0T	SPM8	6	<0.05	GRF	8.5

*FDR, false discovery rate; FWE, family-wise error correction; FWHM, full width at half maximum; GRF, gaussian random field; HS, healthy subjects; MRI, magnetic resonance imaging; NA, not available; ReHo, regional homogeneity; SPM, statistics parameter mapping; T, tesla; TFCE, threshold-free cluster enhancement; TIN, patients with tinnitus; VBM, voxel-based morphometry*.

### Quality Assessment

The quality of all included studies was assessed using an 11-point checklist that was based on those used in previous meta-analyses (Shepherd et al., 2012; Du et al., 2014). The checklist focused on both clinical and demographic characteristics of individual study populations as well as important scanner parameters and methodological details (Table S1) (Shepherd et al., 2012; Du et al., 2014).

Study selection and data extraction and summarization were independently performed in a standardized manner by two investigators (SC and GX); any disagreements were resolved by a third investigator (RS). This meta-analysis adhered to Preferred Reporting Items for Meta-Analyses (PRISMA) guidelines (Moher et al., 2009). This meta-analysis has been registered with the PROSPERO International Prospective Register of Systematic Reviews of the University of York (PROSPERO registration no. CRD42019123399).

### Standard Meta-Analyses of Structural and Functional Abnormalities

Abnormalities in brain structure and spontaneous activity were subjected to the voxel-wise meta-analysis by AES-SDM (https://www.sdmproject.com/software) (Radua et al., 2012a,b).

The extracted peak information was combined to recreate effect-size and variance maps first by means of a Gaussian kernel, which assigned higher effect sizes to voxels closer to the peaks. The FWHM was set at 20 mm in the assignment in order to control for false-positive results (Radua et al., 2012a). Taking sample size, intra-study variability, and between-study heterogeneity into account, study maps were calculated voxel-wise to obtain the random effects mean. Studies with larger sample sizes made a larger contribution because the mean map was weighted by the square root of the sample size of each study. Thresholds were applied using default settings (voxel threshold *P* < 0.005, peak height threshold *z* > 1.00, and cluster size threshold >10 voxels) after calculating for the meta-analysis means (Radua et al., 2012a). Finally, we statistically analyzed the meta-analysis effect-size map by comparing to a null distribution created with a permutation algorithm. A leave-one-out jackknife sensitivity analysis was used to test the reproducibility of VBM and ReHo study findings, which consisted of repeating the mean analysis after systematically removing each study.

In order to exclude potential heterogeneity resulting from different VBM measurement methods, we carried out a subgroup analysis of VBM studies including a scanner (1.5 or 3.0 T scanner) and FWHM (8 and 10 mm). Funnel plots of the peaks of the main findings were performed to check whether the findings might have been driven by few or small studies. Meanwhile, the Egger test was also performed to detect the potential publication bias (Radua and Mataix-Cols, 2009).

### Multimodal Analysis of the Structural and Functional Response

To identify brain areas exhibiting structural and functional alterations in tinnitus patients, the structural and functional data were cross validated in a single meta-analysis map by computing the union of structural and functional *p* values (Radua et al., 2012a).

### Meta-Regression Analysis

To examine any potential effects, a meta-regression analysis weighted by sample size and intra- and between-study variances (Radua et al., 2012a) was performed to evaluate associations between changes in the brain and subject characteristics (age and duration of tinnitus). The probability threshold was decreased to 0.005 to minimize the detection of false associations. Findings for the slope and one of the extremes of the regressor were considered, and those in regions not detected in the main analysis were discarded. Finally, fits obviously driven by an insufficient number of studies were also discarded by inspecting the regression plot (Radua et al., 2012a).

## Results

### Studies Included in the Meta-Analysis

**Figure 1:**
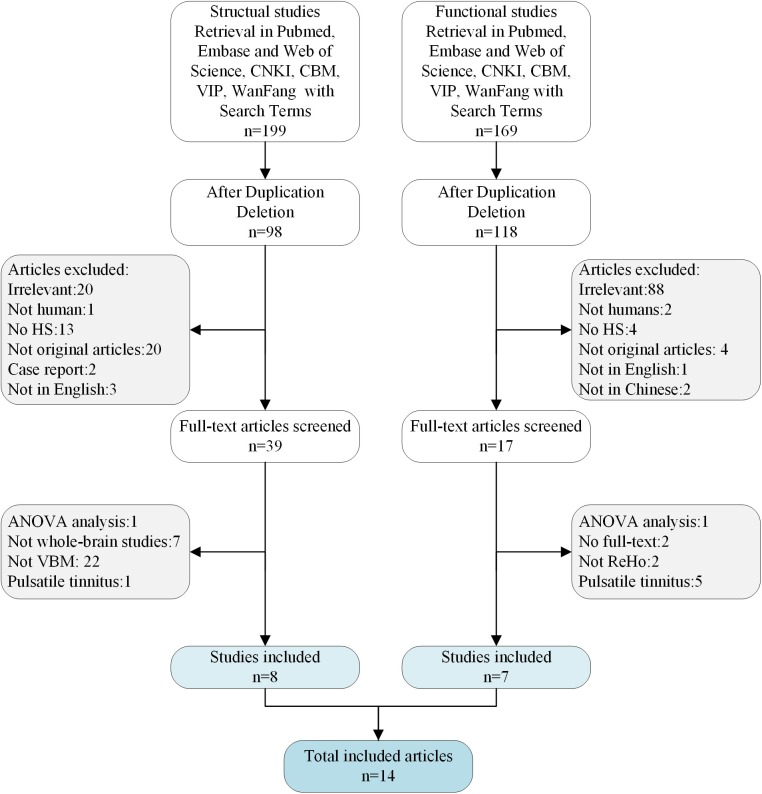
The flow diagram for studies included in the present meta-analysis.

The meta-analysis included 14 studies that met the inclusion criteria (Figure 1). There were eight VBM datasets with a total of 242 patients with tinnitus (107 females; mean age: 48.91 years) and 217 HS (100 females; mean age: 47.66 years), and seven ReHo studies with a total of 130 patients with tinnitus (64 females; mean age: 36.03 years) and 140 HS (70 females; mean age: 37.83 years). All of the patients in these studies were drug naïve and had not undergone a “washout” period prior to MRI scanning. The studies had a mean quality score of 9.73 out of a total possible score of 11, indicating that they were of high quality. There were no significant differences in age or sex ratio between the two groups. Details of the literature search and criteria for article inclusion are shown in Figure 1. The demographic characteristics, clinical variables, scanning method, and significance level of inter-group comparisons in the included studies are shown in Table 1.

### Meta-Analyses of GM Volume Changes

**Figure 2:**
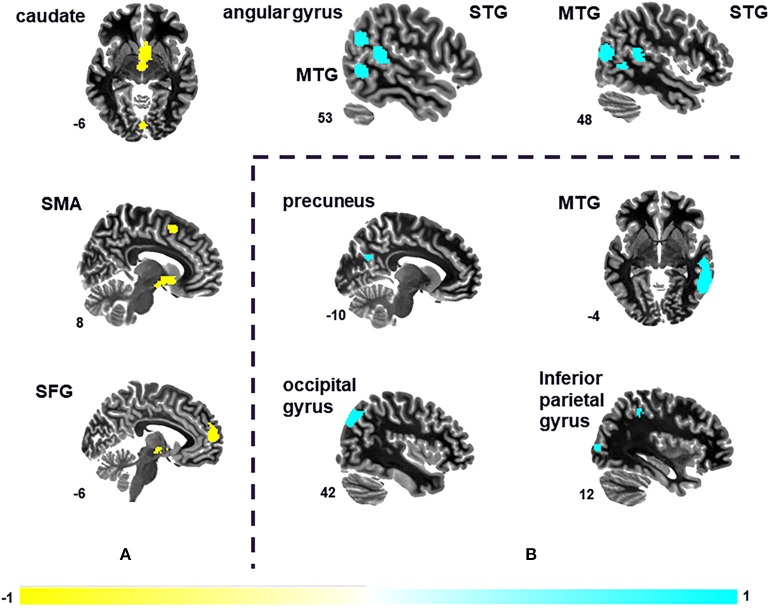
Differences in the brain regions between tinnitus patients and HS. **(A)** Differences in gray matter (GM) volume between tinnitus patients and HS. **(B)** Changes in spontaneous brain activity of tinnitus patients compared with HS. MTG, middle temporal gyrus; SFG, superior frontal gyrus; SMA, supplementary motor area; STG, superior temporal gyrus.

Seven peak foci were reported in this VBM meta-analysis. Patients with tinnitus showed increased GM volume in the right STG (*P* = 0.000, *z* = 1.084), right MTG (*P* = 0.000, *z* = 1.077), left STG (*P* = 0.000, *z* = 1.062), and right angular gyrus (*P* = 0.002, *z* = 1.024), and a decreased volume in the right caudate nucleus (*P* = 0.000, *z* = −1.472), left SFG (*P* = 0.000, *z* = −1.343), and right supplementary motor are (*P* = 0.001, *z* = −1.196) relative to HS (Figure 2A and Table 2). The sensitivity analysis showed that all results above were highly reproducible, as most were preserved in combinations of the dataset (Table S2A).

**Table 2:** VBM brain regions showing GM differences between tinnitus patients and healthy subjects.

	**MNI coordinates**			**SDM *z*-score^a^**	**P value^b^**	**Number of voxels^c^**	**Cluster breakdown (number of voxels)**	**Heterogeneity**	**Sensitivity**
	***x***	***y***	***z***						
**TIN > HS**									
R superior temporal gyrus	50	−40	12	1.084	0.000	254	R superior temporal gyrus, BA22, BA41, BA42 (138)	Yes	7/8
							R arcuate network, posterior segment (64)		
							R superior temporal gyrus, BA21, BA42 (20)		
							Corpus callosum (11)		
R middle temporal gyrus	48	−70	12	1.077	0.000	224	R middle temporal gyrus, BA37, BA39 (163)	Yes	7/8
							R middle occipital gyrus, BA19, BA39 (45)		
L superior temporal gyrus	−46	−34	10	1.062	0.000	203	L superior temporal gyrus, BA41., BA48 (84)	Yes	6/8
							L arcuate network, posterior segment (53)		
							Corpus callosum (32)		
							L Rolandic operculum, BA48 (10)		
R angular gyrus	56	−58	28	1.024	0.002	106	R angular gyrus, BA22, BA39, BA40 (95)	No	6/8
**TIN < HS**									
R caudate nucleus	4	8	−6	−1.472	0.000	599	R striatum (119)	No	6/8
							R olfactory cortex, BA25 (41)		
							R anterior thalamic projections (35)		
							L anterior thalamic projections (30)		
							L olfactory cortex, BA25 (23)		
							Anterior commissure (21)		
							R caudate nucleus, BA25 (14)		
							L caudate nucleus, BA25 (12)		
							Corpus callosum (10)		
L superior frontal gyrus, medial	−8	56	12	−1.343	0.000	358	L superior frontal gyrus, BA10 (261)	No	7/8
							Corpus callosum (80)		
R supplementary motor area	8	10	54	−1.196	0.001	106	Corpus callosum (51)	No	6/8
							R supplementary motor area, BA6 (48)		

a *Peak height threshold: z > 1*.

b *Voxel probability threshold: P < 0.005*.

c *Cluster extent threshold: regions with <10 voxels are not reported in the cluster breakdown*.

*BA, Brodmann area; GM, gray matter; HS, healthy subjects; L, left; MNI, Montreal Neurological Institute; R, right; SDM, signed differential mapping; TIN, tinnitus patients; VBM, voxel-based morphometry*.

### Meta-Analyses of ReHo Abnormalities

A total of four peak foci were reported in the ReHo studies. AES-SDM of the fMRI data revealed hyperactivation in the right MTG (*P* = 0.000, *z* = 1.775), right middle occipital gyrus (*P* = 0.002, *z* = 1.422), left precuneus (*P* = 0.002, *z* = 1.460), and right inferior parietal (excluding supramarginal and angular) gyri (*P* = 0.003, *z* = 1.406) of tinnitus patients relative to HS (Figure 2B and Table 3). The jackknife sensitivity analyses showed that these results were highly reproducible (Table S2B).

**Table 3:** Brain regions showing spontaneous brain activity differences between patients with tinnitus and healthy subjects.

	**MNI coordinates**			**SDM *z*-score^a^**	**P-value^b^**	**Number of voxels^c^**	**Cluster breakdown (number of voxels)**	**Heterogeneity**	**Sensitivity**
	***x***	***y***	***z***						
**TIN > HS**									
R middle temporal gyrus	60	−44	−4	1.775	0.000	442	R middle temporal gyrus, BA21, BA22, BA37 (374)	No	6/7
							R arcuate network, posterior segment (30)		
R middle occipital gyrus	34	−92	6	1.422	0.002	134	R middle occipital gyrus, BA18 (102)	No	4/7
							R inferior network, inferior longitudinal fasciculus (19)		
L precuneus	−10	−64	28	1.460	0.002	127	L median network, cingulum (69)	No	5/7
							L precuneus, BA23 (32)		
							L cuneus cortex (14)		
R inferior parietal (excluding supramarginal and angular) gyri	34	−44	46	1.406	0.003	38	R inferior parietal (excluding supramarginal and angular) gyri, BA40 (17)	No	5/7

a *Peak height threshold: z > 1*.

b *Voxel probability threshold: P < 0.005*.

c *Cluster extent threshold: regions with <10 voxels are not reported in the cluster breakdown*.

*BA, Brodmann area; HS, healthy subjects; L, left; MNI, Montreal Neurological Institute; R, right; SDM, signed differential mapping; TIN, patients with tinnitus*.

### Multimodal Analysis of GM Volume and Brain Response

We summarized our findings into a single meta-analytic map in order to identify regions showing abnormalities in both VBM and ReHo studies. The multimodal analysis revealed significant differences in the right MTG (*P* < 0.0025) of patients with tinnitus relative to HS.

### Heterogeneity Analysis and Publication Bias

The heterogeneity analysis indicated significant heterogeneity among the VBM studies with altered GM volume in the right STG, right MTG, and left STG (*P* < 0.005) (Table S3) and showed insignificant heterogeneity among the ReHo studies. We conducted the funnel plots and Egger tests, although there were too few VBM and ReHo studies for evaluation of publication bias in the meta-analysis, which requires at least 10 studies. The funnel plots demonstrated that the main findings were driven by at least seven VBM studies and six ReHo studies, respectively (Figure S1). Analysis of publication bias revealed that the Egger tests were insignificant in the peaks of the altered brain regions in the VBM meta-analysis (*P* = 0.715) and the ReHo meta-analysis (*P* = 0.990).

### Subgroup Analysis

A subgroup analysis of VBM studies using different FWHM values (8 mm/10 mm) yielded no significant differences. The subgroup analysis of VBM studies using different scanners (1.5/3.0 T) revealed structural abnormality in the left medial SFG and right supplementary motor area of patients with tinnitus (Table S4).

### Meta-Regression

Although it is not recommended for fewer than nine studies (Radua and Mataix-Cols, 2009), we carried out a meta-regression analysis to examine potential confounding variables (mean age and disease duration). The mean age of patients was associated with increased GM volume in the bilateral STG, right MTG, right angular gyrus in VBM studies (Table S5A). Moreover, the mean age of patients was associated with hyperactivation in the right MTG and right middle occipital gyrus in ReHo studies (Table S5B). The meta-regression of disease duration showed insignificance among the brain regions in VBM studies and ReHo studies.

## Discussion

This is the first multimodal neuroimaging meta-analysis investigating the neural substrates of tinnitus by combining information from whole-brain VBM studies of GM volume and ReHo studies of spontaneous brain activity.

The main findings were that GM volume was increased in bilateral STG, the right MTG, and right angular gyrus and decreased in the right caudate nucleus, left medial SFG, and right supplementary motor area of patients with tinnitus compared with HS (Figure 2A and Table 2). Additionally, tinnitus was associated with hyperactivation in the right MTG, right middle occipital gyrus, left precuneus, and right inferior parietal (excluding supramarginal and angular) gyri (Figure 2B and Table 3). These results remained unchanged when each study was removed in the jackknife sensitivity analysis.

Abnormalities are more frequently reported in the STG and MTG than in other parts of the auditory cortex in neuroimaging studies of tinnitus (Boyen et al., 2013; Chen et al., 2014; Han et al., 2018a). The temporal gyrus is critical for auditory processing that includes simple auditory stimulus processing and semantic memory (May and Tiitinen, 2010; Recasens et al., 2014), and is a connection in the hierarchical structure of the primary auditory cortex and frontal lobe (Ishishita et al., 2019). Our results revealed abnormal brain responses in this region, suggesting their involvement in tinnitus. Prolonged exposure to an internal sound such as a tone and/or noise is associated with increased GM volume in cortical regions of auditory processing (Boyen et al., 2013).

Tinnitus patients often exhibit varying degrees of hearing impairment. Hearing loss patients with or without tinnitus use more contextual information from semantic memory to maintain normal communication, which may result in increased GM volume of the auditory association area (Boyen et al., 2013). However, a recent neuroimaging study did not find any differences in the temporal gyrus between recent-onset patients with tinnitus with mild hearing impairment and HS (Krick et al., 2015). As the present meta-analysis included patients with tinnitus as well as those without hearing loss, we were unable to perform a regression analysis of hearing loss severity. In addition, differences in the temporal gyrus have been reported between long-term and recent-onset patients with tinnitus (Schmidt et al., 2018). Although this implies that the duration of tinnitus alters the temporal gyrus, we did not find any correlation between these two variables in this meta-analysis. Furthermore, some studies have shown increased neuronal activity in the right hemisphere—especially the right MTG—in tinnitus (Mirz et al., 1999; Chen et al., 2014), whereas others have reported left lateralization (Langguth et al., 2006; Geven et al., 2014). Therefore, although the right MTG involvement found in this study suggests right lateralization, most patients exhibited unilateral or bilateral tinnitus, making it difficult to conclude that tinnitus has a unilateral tendency.

Tinnitus sensation representations generated or expressed in auditory cortex are necessary (Lanting et al., 2009) but not sufficient to account for suffering from tinnitus. Central auditory system hypotheses of tinnitus genesis have been proposed to account for the discrepancy between audiometric profile and tinnitus perceptual attributes, including thalamocortical dysrhythmia in frequencies (Llinas et al., 1999; Weisz et al., 2007), the striatal gating model (Larson and Cheung, 2012), etc. Decreased GM volume of caudate nucleus has been found in patients with tinnitus in this meta-analysis, supporting that the striatal gating model might be involved in tinnitus genesis. The caudate nucleus has been found be projected from the superior temporal cortex and rostral and midportion aspects of association cortex on the superior temporal gyrus in monkeys and cats (Reale and Imig, 1983; Yeterian and Pandya, 1998). This nucleus is hypothesized to act as a gating mechanism for auditory phantom percepts, which can suppress the enduring tinnitus loudness (Larson and Cheung, 2013). The requisite cortico-striatal neural circuitry is in place for dysfunction striatal connectivity to enable perception of auditory phantoms (Larson and Cheung, 2012; Hinkley et al., 2015).

We also found decreased GM volume of SFG in patients with tinnitus. This brain region plays an important role in executive function and emotion processing and attention, in contrast to the sensory processing that is affected in chronic tinnitus (Mirz et al., 2000; Weisz et al., 2004). Results from resting-state fMRI studies indicate that SFG is the main cortical area affected by tinnitus (Chen et al., 2014, 2016). Moreover, disrupted functional connectivity between the SFG and amygdala has been observed in patients, suggesting that executive control of attention network in the SFG enhances the negative attributes of tinnitus contributed by the amygdala (Chen et al., 2017). A decreased SFG GM volume has been attributed to peripheral hearing loss (Husain et al., 2011; Boyen et al., 2013). In our meta-analysis, there was no available uniform audiometric data reported in the included studies, making it difficult to distinguish between the effects of hearing impairment and tinnitus on SFG alteration.

Besides auditory brain regions, auditory spatial information is also processed by the visual centers of the brain (Bedny et al., 2010; Fiehler and Rosler, 2010). Increased activity in the right middle occipital gyrus of patients with tinnitus were observed in the current meta-analysis. This brain region participates in visual processing, which may be processed cross-modally in phantom auditory perception (Murray et al., 2005). Furthermore, the increased GM volume of angular and hyperactivation of precuneus were revealed in this meta-analysis. Interestingly, the MTG, angular gyrus, and precuneus also belong to the default mode network (DMN), which is most active at rest (Raichle et al., 2001; Mantini et al., 2007). These aberrant neural activities may be responsible for the dysfunction of DMN in patients with tinnitus, although the source or their type within specific DMN regions due to tinnitus remains unclear.

The analysis of heterogeneity showed that findings from structural neuroimaging studies were inconsistent, while the subgroup analysis using a different scanner revealed different structural abnormalities in the left medial SFG and right supplementary motor area among patients with tinnitus. In addition, the mean age of patients was associated with increased GM volume in the bilateral STG, right MTG in VBM studies. This variability could be due to small sample sizes, demographic characteristics, and methodological differences (e.g., in the scanner) among studies. One study reported differences in GM of various brain regions between patients with mild and more severe long-term tinnitus (Schmidt et al., 2018). We did not perform meta-regression of tinnitus severity in this study as the different questionnaires used to assess tinnitus symptoms were not uniform; however, the severity of tinnitus may also contribute to the observed heterogeneity.

The multimodal analysis revealed significant differences in the right MTG of patients with tinnitus and HS. The temporal cortex—specifically the MTG—was found to be altered in patients with tinnitus (Mirz et al., 1999; May and Tiitinen, 2010; Chen et al., 2014; Recasens et al., 2014), suggesting that this brain area is closely associated with tinnitus development (Farhadi et al., 2010).

The present study had some limitations. First, a relatively limited number of datasets were analyzed owing to the exclusion criteria. There were only 14 studies included totally; the results of this meta-analyses were preliminary. Second, patients with tinnitus, both with and without hearing loss, were included, which precluded a regression analysis of hearing loss severity.

In conclusion, the results of our meta-analysis revealed abnormalities not only in auditory areas but also in the caudate nucleus, which suggests the involvement of the cortico-striatal circuits in the neuropathology of tinnitus.

## Data Availability Statement

The datasets analyzed in the current study are available from the corresponding author upon reasonable request.

## Author Contributions

RS, SC, and FL contributed to study conception and design and conceived the data analysis strategy. SC, GX, and JZ acquired the data. SC, GX, and RS collated and analyzed the data. SC, GX, and JZ drafted the manuscript. YQ, ZL, ZH, TY, PM, RS, and FL discussed, read, and revised the manuscript. All authors approved the publication of this manuscript.

### Conflict of Interest

The authors declare that the research was conducted in the absence of any commercial or financial relationships that could be construed as a potential conflict of interest.
