# Supplementary material for: A Multimodal Meta-Analysis of Structural and Functional Changes in the Brain of Tinnitus
Source: Front Hum Neurosci. 2020 Feb 25;14:28. doi: 10.3389/fnhum.2020.00028 (PMC7053535; doi:10.3389/fnhum.2020.00028)
Supplement: Supplementary file 1 [file Data_Sheet_1.docx]

# Table S1: Quality Assessment Checklist: 1 point per criterion fully satisfied, 0.5 for partially satisfied, 0 for otherwise

| **Category 1: Subjects**  **Score** (0/0.5/1) |
| --- |
| 1 Patients were evaluated prospectively, specific diagnostic criteria were applied, and demographic data was reported |
| 2 Healthy comparison subjects were evaluated prospectively, psychiatric and medical illnesses were excluded |
| 3 Important variables (e.g. age, gender, illness duration, onset time, medication status, comorbidity, severity of illness) were checked, either by stratification or statistically |
| 4 Sample size per group > 10 |
| **Category 2: Methods for image acquisition and analysis** |
| 5 Magnet strength ≥1.5T |
| 6 For VBM studies, MRI slice thickness ≤2 mm; for resting-state functional MRI studies, whole brain analysis was automated with no *a* *priori* regional selection |
| 7 Coordinates reported in a standard space |
| 8 The imaging technique processing was described clearly enough to be reproduced |
| 9 Measurements were clearly described clearly enough to be reproduced |
| **Category 3: Results and conclusions** |
| 10 Statistical parameters were provided for significant, and important non-significant, differences |
| 11 Conclusions were consistent with the results obtained and the limitations were discussed |
| **TOTAL**  /11 |

Abbreviations: MRI, magnetic resonance imaging; T, tesla; VBM, voxel-based morphometry.

# Table S2A: Sensitivity analysis of VBM meta-analysis

|  | Increased GM volume Regions | | |  |  | Decreased GM volume Regions | | |
| --- | --- | --- | --- | --- | --- | --- | --- | --- |
| **VBM Studies** | R superior temporal gyrus | R middle temporal gyrus | L superior temporal gyrus | R angular gyrus |  | R caudate nucleus | L superior frontal gyrus, medial | R supplementary motor area |
| Muhlau et al 2006 | Yes | Yes | Yes | Yes |  | No | Yes | Yes |
| Landgrebe et al 2009 | Yes | Yes | Yes | Yes |  | Yes | Yes | Yes |
| Boyen et al 2013 | No | No | No | No |  | No | No | Yes |
| Melcher et al 2013 | Yes | Yes | Yes | Yes |  | Yes | Yes | Yes |
| Krick et al 2015 | Yes | Yes | Yes | Yes |  | Yes | Yes | No |
| Allan et al 2016 | Yes | Yes | No | No |  | Yes | Yes | Yes |
| Schmidt et al 2018 | Yes | Yes | Yes | Yes |  | Yes | Yes | Yes |
| Han et al 2018b | Yes | Yes | Yes | Yes |  | Yes | Yes | No |

Abbreviations: GM, gray matter; L, left; R, right; VBM, voxel-based morphometry.

# Table S2B: Sensitivity analysis of ReHo meta-analysis

|  | Increased spontaneous activity regions | | | |
| --- | --- | --- | --- | --- |
| **ReHo Studies** | R middle temporal gyrus | R middle occipital gyrus | L precuneus | R inferior parietal (excluding supramarginal and angular) gyri |
| Jin et al 2011 | Yes | Yes | Yes | No |
| Yang et al 2014 | Yes | Yes | Yes | Yes |
| Chen et al 2015 | Yes | No | Yes | No |
| Cai et al 2017 | Yes | No | No | Yes |
| Han et al 2018a | No | No | Yes | Yes |
| Han et al 2018b | Yes | Yes | Yes | Yes |
| Fan et al 2018 | Yes | Yes | No | Yes |

Abbreviations: L, left; R, right; ReHo, regional homogeneity.

# Table S3: Heterogeneity of altered brain regions between Tinnitus Patients and Healthy Subjects in VBM studies

| Region | MNI coordinate | | | SDM z-score ^(a)^ | p-value ^(b)^ | Number of voxels ^(c)^ |
| --- | --- | --- | --- | --- | --- | --- |
|  | x | y | z |  |  |  |
| R superior temporal gyrus | 50 | -40 | 12 | 3.903 | 0.000 | 124 |
| R middle temporal gyrus | 48 | -70 | 12 | 3.777 | 0.000 | 78 |
| L superior temporal gyrus | -46 | -34 | 10 | 3.463 | 0.000 | 181 |

^a^Peak height threshold: z > 1;

^b^Voxel probability threshold: p < 0.005;

^c^Cluster extent threshold: Regions with less than 10 voxels are not reported in the cluster breakdown.

Abbreviations: L, left; MNI, montreal neurological institute; R, right; SDM, signed differential mapping; VBM, voxel-based morphometry.

# Table S4: Regional Differences between 1.5T Scanner and 3.0T Scanner Derived from VBM Studies

| Region | MNI coordinate | | | SDM z-score ^(a)^ | p-value ^(b)^ | number of voxels ^(c)^ | cluster breakdown (number of voxels) |
| --- | --- | --- | --- | --- | --- | --- | --- |
|  | x | y | z |  |  |  |  |
| **TIN < HS** |  |  |  |  |  |  |  |
| L superior frontal gyrus, medial | -8 | 56 | 8 | -1.736 | 0.000 | 429 | L superior frontal gyrus, medial, BA10 (273) |
|  |  |  |  |  |  |  | Corpus callosum (95) |
|  |  |  |  |  |  |  | R superior frontal gyrus, medial, BA10 (22) |
|  |  |  |  |  |  |  | L superior frontal gyrus, dorsolateral, BA10 (22) |
| R supplementary motor area | 6 | 10 | 54 | -1.543 | 0.000 | 349 | R supplementary motor area, BA6, BA8, BA32 (230) |
|  |  |  |  |  |  |  | Corpus callosum (101) |
|  |  |  |  |  |  |  | R superior frontal gyrus, dorsolateral, BA8 (10) |

^a^Peak height threshold: z > 1;

^b^Voxel probability threshold: p < 0.005;

^c^Cluster extent threshold: Regions with less than 10 voxels are not reported in the cluster breakdown.

Abbreviations: HS, healthy subjects; L, left; MNI, montreal neurological institute; R, right; SDM, signed differential mapping; T, tesla; TIN, tinnitus patients; VBM, voxel-based morphometry.

# Table S5A Meta-regression analysis for structural abnormalities in tinnitus patients.

| Region | MNI coordinate | | | SDM z-score ^(a)^ | p-value ^(b)^ | number of voxels ^(c)^ |
| --- | --- | --- | --- | --- | --- | --- |
|  | x | y | z |  |  |  |
| **Effect of age** |  |  |  |  |  |  |
| R superior temporal gyrus | 50 | -40 | 12 | 1.072 | 0.001 | 162 |
| R middle temporal gyrus | 48 | -70 | 12 | 1.065 | 0.001 | 121 |
| L superior temporal gyrus | -48 | -34 | 12 | 1.053 | 0.001 | 344 |
| R angular gyrus | 56 | -58 | 28 | 1.002 | 0.003 | 32 |

^a^Peak height threshold: z > 1;

^b^Voxel probability threshold: p < 0.005;

^c^Cluster extent threshold: Regions with less than 10 voxels are not reported in the cluster breakdown.

Abbreviations: L, left; MNI, montreal neurological institute; R, right; SDM, signed differential mapping.

# Table S5B Meta-regression analysis for functional abnormalities in tinnitus patients.

| Region | MNI coordinate | | | SDM z-score ^(a)^ | p-value ^(b)^ | number of voxels ^(c)^ |
| --- | --- | --- | --- | --- | --- | --- |
|  | x | y | z |  |  |  |
| **Effect of age** |  |  |  |  |  |  |
| R middle temporal gyrus | 60 | -40 | -4 | 2.155 | 0.000 | 655 |
| R middle occipital gyrus | 34 | -90 | 0 | 1.808 | 0.001 | 205 |

^a^Peak height threshold: z > 1;

^b^Voxel probability threshold: p < 0.005;

^c^Cluster extent threshold: Regions with less than 10 voxels are not reported in the cluster breakdown.

Abbreviations: MNI, montreal neurological institute; R, right; SDM, signed differential mapping.

# Figure S1. Funnel plots of VBM studies and ReHo studies





**Figure S1.** Funnel plots of VBM studies and ReHo studies. **A.** Funnel plot of VBM studies. **B.** Funnel plot of ReHo studies.

Abbreviations: ReHo, regional homogeneity; VBM, voxel-based morphometry.
